# Rare malignant neoplasm of the esophagus: current status and future perspectives

**DOI:** 10.1093/jjco/hyad144

**Published:** 2023-10-19

**Authors:** Yuri Yoshinami, Erica Nishimura, Taisuke Hosokai, Shun Yamamoto, Satoru Matsuda, Motoo Nomura, Hirofumi Kawakubo, Ken Kato, Yuko Kitagawa

**Affiliations:** Department of Head and Neck, Esophageal Medical Oncology, National Cancer Center Hospital, Tokyo, Japan; Department of Surgery, Keio University School of Medicine, Tokyo, Japan; Department of Clinical Oncology, Kyoto University Hospital, Kyoto, Japan; Department of Head and Neck, Esophageal Medical Oncology, National Cancer Center Hospital, Tokyo, Japan; Department of Surgery, Keio University School of Medicine, Tokyo, Japan; Department of Clinical Oncology, Kyoto University Hospital, Kyoto, Japan; Department of Surgery, Keio University School of Medicine, Tokyo, Japan; Department of Head and Neck, Esophageal Medical Oncology, National Cancer Center Hospital, Tokyo, Japan; Department of Surgery, Keio University School of Medicine, Tokyo, Japan

**Keywords:** rare cancer, neuroendocrine neoplasm, gastrointestinal stromal tumor, carcinosarcoma, primary malignant melanoma of esophagus

## Abstract

Esophageal cancer is common worldwide, including in Japan, and its major histological subtype is squamous cell carcinoma. However, there are some rare esophageal cancers, including neuroendocrine neoplasm, gastrointestinal stromal tumor, carcinosarcoma and malignant melanoma. The biological and clinical features of these cancers differ from those of esophageal squamous cell carcinoma. Therefore, different treatment strategies are needed for these cancers but are based on limited evidence. Neuroendocrine neoplasm is mainly divided into neuroendocrine tumor and neuroendocrine carcinoma by differentiation and the Ki-67 proliferation index or mitotic index. Epidemiologically, the majority of esophageal neuroendocrine neoplasms are neuroendocrine carcinoma. The treatment of neuroendocrine carcinoma is similar to that of small cell lung cancer, which has similar morphological and biological features. Gastrointestinal stromal tumor is known to be associated with alterations in the c-KIT and platelet-derived growth factor receptor genes and, if resectable, is treated in accordance with the modified Fletcher classification. Carcinosarcoma is generally resistant to both chemotherapy and radiotherapy and requires multimodal treatments such as surgery plus chemotherapy to achieve cure. Primary malignant melanoma is resistant to cytotoxic chemotherapy, but immune checkpoint inhibitors have recently demonstrated efficacy for malignant melanoma of the esophagus. This review focuses on the current status and future perspectives for rare cancer of the esophagus.

## Teaser

Rare esophageal cancers, including neuroendocrine neoplasm, gastrointestinal stromal tumor, carcinosarcoma and melanoma, differ from esophageal squamous cell carcinoma and require treatment strategies with a limited evidence base.

## Introduction

In 2018, the number of esophageal cancer (EC) cases worldwide was estimated as 572 000, and the deaths of deaths due to EC was estimated as 509 000. Globally, EC is the seventh most common cancer in terms of incidence and the sixth in terms of mortality (1).

One of the major histological subtypes of EC is squamous cell carcinoma. However, there are some rare ECs, which are defined as cancers with an incidence rate of < 6 per 100 000 persons per year and include neuroendocrine neoplasm (NEN), gastrointestinal stromal tumor (GIST), esophageal carcinosarcoma (ESC) and primary malignant melanoma of the esophagus (PMME) (2). The biological characteristics of these cancers are different from those of the major histological subtypes, namely, esophageal squamous cell carcinoma and adenocarcinoma. Therefore, different treatment strategies are needed for these rare cancers based on limited evidence.

This article discusses the current status of these rare cancers, including NEN, GIST, ESC and PMME, and outlines future perspectives in the form of a narrative review by medical and surgical oncologists.

## Neuroendocrine neoplasm

### Epidemiology and diagnosis

NEN is defined as a tumor arising from or differentiating into neuroendocrine cells present in various tissues and histologically expressing neuroendocrine markers such as chromogranin A, neuron-specific enolase and synaptophysin. A very rare cancer, NEN can occur throughout the body but is most commonly of gastroenteropancreatic (GEP) origin. According to the World Health Organization classification (3,4), pathological classification is important and morphological differentiation (highly/poorly differentiated) and cell proliferative potential (grade 1, 2 or 3) as assessed by nuclear fission imaging and the Ki-67 proliferation index. NEN is classified into two major types: highly differentiated neuroendocrine tumor (NET) and poorly differentiated neuroendocrine carcinoma (NEC). In Japan, the number of new cases per year (per 100 000 population) was reported to be 0.70 for pancreatic NEN and 2.84 for gastrointestinal NEN (5).

Esophageal neuroendocrine neoplasm (E-NEN) is very rare and has been reported to account for 0.03% of esophageal malignancies (6) while esophageal neuroendocrine carcinoma (E-NEC) has been found to account for 6–56% of primary gastrointestinal NEC (7,8). NEC accounts for 0.3–1.0% of esophageal malignancies and is more common in Asian countries than in Western countries (6,9). In contrast, esophageal neuroendocrine tumor (E-NET) was found to have a very low frequency of 0.04% in a large cohort of patients with gastrointestinal NET in the US and Europe, and has never been studied in a large number of cases (10). Therefore, this review focusses mainly on E-NEC.

E-NEC is more common in men than in women and has a predilection for those aged 50–70 years. No specific risk factors have been identified. The primary site of E-NEC is usually the middle esophagus, and it is often in an advanced stage with lymph node metastasis at the time of diagnosis. Distant metastases are usually located in the liver, lung and bone, while brain metastases are relatively rare (11,12). E-NEC has an extremely poor prognosis, with a reported median survival of 4.2 to 18.5 months (13).

The 2019 WHO classification defines mixed neuroendocrine-non-neuroendocrine neoplasms (MiNEN) as those containing more than 30% each of NEN and other components (3,4). MiNEN has been reported to occur not only in the esophagus but also in gastrointestinal organs such as the stomach, Vater’s papilla and colon, but cases are extremely limited (14). The median survival of MiNEN is approximately 20 months, suggesting a better prognosis than pure NEC (4). MiNEN is expected to be related to the presence of primary tumor component such as adenocarcinoma, squamous cell carcinoma and treatments for MiNEN is usually performed in accordance with treatments for NEN (15).

### Treatment of local/locoregional disease

The European Neuroendocrine Tumor Society (16) and European Society for Medical Oncology (17) guidelines for the management of GEP-NEC recommend platinum-based doublet therapy in accordance with the treatment of small cell lung cancer (SCLC), which is morphologically and biologically similar to NEC.

Surgical resection might be considered in resectable cases. However, the National Comprehensive Cancer Network (NCCN) guidelines recommend a combination of chemotherapy and radiotherapy (18), although no standard treatment has been established for patients with locally advanced E-NEC.

Kikuchi et al. (19) reported that local treatments for patients with clinical stage I-III E-NEC achieved a median survival time of 9 months in those who underwent surgery alone, 31 months in those who underwent surgery plus adjuvant chemotherapy, and 25 months in those who received neoadjuvant chemotherapy plus surgery. Overall survival (OS) was significantly longer in the adjuvant chemotherapy and neoadjuvant chemotherapy groups than in the surgery only group. However, there was no significant difference in OS between the adjuvant chemotherapy and neoadjuvant chemotherapy groups or between the chemotherapy ± radiation and neoadjuvant chemotherapy groups. Neoadjuvant chemotherapy might be recommended for patients with resectable locally advanced E-NEC (20). However, there is little evidence to guide the treatment of patients with E-NEC.

In addition, the indications for chemoradiotherapy need to be considered. Definitive chemoradiotherapy showed promising efficacy in patients with locally advanced E-NEC in China (21). Honma et al. (22) retrospectively investigated clinical outcomes, feasibility and prognostic factors in Japanese patients with locally advanced E-NEC treated with definitive chemoradiotherapy, which consisted of radiotherapy (60 Gy/30 fractions) combined with platinum plus etoposide or cisplatin plus 5-fluorouracil. The overall response rate was 86.4% and the clinical complete remission rate was 77.3%, with a median progression-free survival of 12.7 months and a median survival time of 37.5 months. The findings of that study suggest that definitive chemoradiotherapy may be an important treatment option for patients with locally advanced E-NEC.

### Summary of treatment for local/locoregional E-NEC

For locally advanced E-NEC, surgery plus neoadjuvant or adjuvant chemotherapy, and definitive chemoradiotherapy are important treatments options, and these therapies might be widely conducted in the clinical practice. However, the optimal treatment for locally advanced E-NEC is still unclear, further investigations are needed.

#### Treatment of metastatic/recurrent disease

Patients with metastatic or recurrent disease are treated with palliative chemotherapy as in SCLC. The 2019 Japanese Guidelines for Gastroenteropancreatic Neuroendocrine Neoplasms recommend combination therapy that includes platinum-based agents and etoposide or irinotecan (20) while the NCCN guidelines recommend etoposide plus cisplatin (EP), etoposide plus carboplatin (EC), irinotecan plus cisplatin (IP), irinotecan plus carboplatin, FOLFOX, FOLFIRI, FOLFIRINOX or temozolomide plus capecitabine (18). However, there is no evidence for an optimal first-line regimen in patients with metastatic or recurrent NEC based on the results of randomised controlled trials.

Therefore, the Japanese Clinical Oncology Group performed a multicenter, open-label, phase III randomised controlled trial (JCOG1213, TOPIC-NEC) to determine whether EP or IP was a more effective regimen in terms of OS as a primary endpoint in patients with advanced NEC of the digestive system, including E-NEC (23). This trial enrolled chemotherapy-naive patients aged 20–75 years who had recurrent or unresectable NEC (according to the 2010 World Health Organization classification system) arising from the gastrointestinal tract, hepatobiliary system or pancreas.

A total of 170 patients were enrolled in the trial. The median OS was reported to be 12.5 months (95% confidence interval [CI] 10.3–15.7) in the EP group and 10.9 months (95% CI 8.9–13.1) in the IP group (hazard ratio [HR] 1.04, 95% CI 0.79–1.37, p = 0.80). The median PFS was reported to be 5.6 months (95% CI 4.1–6.9) in the EP group and 5.1 months (95% CI 3.3–5.7) in the IP group (HR 1.06, 95% CI 0.78–1.45). The primary analysis revealed no statistically significant difference in OS between the groups; 15.5% of the tumors in the EP group and 9.3% of those in the IP group were E-NEC, and subgroup analysis revealed no significant differences between the two groups. With regard to grade 3 or 4 adverse events (AEs), neutropenia occurred in 91.5% of patients in the EP group and in 30.5% of those in the IP group, and febrile neutropenia in 26.8 and 12.2%, respectively. Therefore, primary prophylactic administration of granulocyte colony-stimulating factor (G-CSF) was recommended in the EP group. However, in terms of all grades of AEs, diarrhea was more frequent in the IP group than in the EP group (47.6% vs. 23.2%). The results of this trial indicated that both EP and IP can be used as standard first-line chemotherapy in patients with advanced gastrointestinal NEC.

EC therapy is also a treatment option for patients with metastatic/recurrent NEC who are intolerant to cisplatin. Carboplatin does not need hydration, which makes it easier to use in the elderly and in patients with renal or cardiac failure. Sorbye et al. (7) reported an objective response rate (ORR) of 30% and a median OS of 11 months, which is comparable with the ORR of 31% and the median OS of 12 months in a similar report on EP therapy. Imai et al. (24) reported an ORR of 47.4%, a PFS of 7.0 months, and an OS of 12.7 months in 19 patients with extrapulmonary NEC, with major AEs including leukopenia (73.7%), neutropenia (78.9%), anemia (31.6%) and thrombocytopenia (26.3%).

Second-line regimens used in the treatment of SCLC are often used in NEC without using first-line drug therapy but have not been standardised. Amrubicin is generally used as second-line treatment for SCLC. However, there have been few reports on its use in E-NEC, apart from one study that reported an ORR of 4% and a PFS of 1.9 months (9). Furthermore, some reports suggested that 5-fluorouracil and levofolinate, oxaliplatin (FOLFOX) therapy (25) and 5-fluorouracil and levofolinate, irinotecan (FOLFIRI) therapy (26) might be useful after second-line treatment.

### Summary of metastatic/recurrent E-NEC

For metastatic or recurrent E-NEC, EP and IP are recommended as the first-line standard treatments. However, EC might be an important option for metastatic or recurrent E-NEC patients intolerant to cisplatin. After the first-line, some articles were reported efficacy of amrubicin and FOLFOX, FOLFIRI, the efficacy was stull limited (Table 1) Therefore, developments of effective salvage-line treatments are needed.

**Table 1:** Previous reports on palliative chemotherapy for neuroendocrine carcinoma

**Authors**	**Line**	**N**	**Regimen**	**ORR (%)**	**Median PFS (month)**	**Median OS (month)**
Morizane et al. (23)	1	170	EP IP	54.5 52.5	5.6 5.1	12.5 10.9
Sorbye et al. (7)	1	252	EP (a) EC (b) Carboplatin/Etoposide/Vincristine (c)	(a)31 (b)30 (c)44	(a)4 (b)4 (c)4	(a)12 (b)11 (c)10
Yamaguchi et al. (9)	2	25	Amrubicin	4	1.9	8.3
Hadoux et al. (25)	2	20	FOLFOX	29	4.5	9.9
Hentic et al. (26)	2	19	FOLFIRI	31	4	18

Abbreviations: ORR, objective response rate; PFS, progression-free survival; OS, overall survival; EP, etoposide plus cisplatin; IP, irinotecan plus cisplatin; EC, etoposide plus carboplatin; FOLFOX, 5-FU plus levofolinate, oxaliplatin; FOLFIRI, 5-FU plus levofolinate, irinotecan

### Future perspectives

Treatments for patients for advanced NEC are similar to those used in SCLC. Recently, immune checkpoint inhibitors have been developed for use in a variety of cancers, including SCLC. The CASPIAN trial was a global, randomised, phase III trial that compared durvalumab plus EP/EC with EP/EC as first-line treatment for advanced SCLC (27). Interim analysis of the CASPIAN trial showed that median OS which was primary endpoint was 13.0 months (95% CI 11.5–14.8) in the durvalumab arm and 10.3 months (95% CI 9.311.2) in the chemotherapy arm (HR 0.73, 95% CI 0.59–0.91). Furthermore, the randomised, double-blind, phase III IMpower133 trial compared atezolizumab plus EC therapy with EC therapy as first-line treatment for advanced SCLC (28). The primary endpoints were OS and PFS, median OS was 12.3 months (95% CI 10.8–15.9) in the atezolizumab arm and 10.3 months (95% CI 9.3–11.3) in the chemotherapy arm (HR 0.70, 95% CI 0.54–0.91) while PFS was 5.2 months (95% CI 4.4–5.6) and 4.3 months (95% CI 4.2–4.5), respectively (HR 0.77, 95% CI 0.62–0.96). Based on the results of these trials, combination platinum therapy plus a PD-L1 inhibitor is recommended for SCLC. Therefore, regimens combining EP or EC therapy with anti-PD-L1 antibody agent might be effective in primary gastrointestinal NEC. However, at present, the efficacy and safety data are only preliminary.

There are currently no randomised controlled trials in NEC. A phase II trial (NCT03901378) of pembrolizumab in combination with EC or EP therapy in primary high-grade gastrointestinal NEC is presently underway in the US. Other ongoing trials includes a phase II trial of avelumab after failure of EP therapy in Korea (NCT03147404) and a phase II trial of avelumab in NEC after chemotherapy in Germany (NCT03352934).

Many clinical trials of combination therapies with cytotoxic agents and immunotherapeutic agents are in progress for each treatment line. We expect therapeutic outcomes for E-NEC to be improved in the near future.

## Gastrointestinal stromal tumor

### Epidemiology and diagnosis

GIST is the most common mesenchymal neoplasm arising from the digestive tract and has an annual incidence of 7–20 per million (29). These tumors arise primarily in the stomach (50%), followed by the small intestine (30%) and the colon or rectum (5%) (30). Esophageal GIST is a very rare entity and represents <1% of all cases (31). Given the rarity of esophageal GIST, there is limited information on its clinical features and no clear recommendation for optimal treatment, including surgical management. Esophageal GIST is most commonly located in the lower esophagus, followed by the middle esophagus, while GIST in the upper esophagus is rare (32). This might be explained by the distribution of the intestinal cells of Cajal, which are known to be precursors of GIST (33). Considering that these cells are abundant in the lower esophagus and rare in the upper segment (34), it seems reasonable that they are most commonly found in the lower esophagus. The symptoms of GIST vary according to tumor size and location. Dysphagia is the most frequent symptom (found in 36–51% of cases), followed by weight loss (20%), chest pain (8–15%) and bleeding (35). In general, the modified Fletcher classification system has been applied to assess the malignancy of GIST based on the primary site, tumor diameter, and the presence or absence of capsular rupture (36). Certain gene mutations have also been reported to be related to the prognosis. Researchers has discovered that about 80% of GISTs have a mutation in KIT and 5–10% have a mutation in platelet-derived growth factor receptor alpha (PDGFRA) (33,37). KIT and PDGFRA mutations can predict the response to tyrosine kinase inhibitors, such as imatinib (38). While KIT exon 11 and 9 mutant GISTs are sensitive to imatinib, PDGFRA exon 18 D842V mutant GISTs are known to be resistant (38).

### Treatments and future perspectives

Complete surgical resection is usually considered for localised GIST (39). However, because GIST rarely metastasizes to lymph nodes, routine lymphadenectomy is not recommended (40). Although gastric and intestinal GISTs can be removed by segmental or wedge resection, esophageal GIST is essentially limited to enucleation or esophagectomy because of its anatomical peculiarity (41). Successful R0 resection has been shown to afford a good prognosis (42). In view of the risk of rupture, esophagectomy should be considered to achieve complete resection and a negative margin. However, enucleation can be a treatment option in patients with comorbidities when the tumor is small (41,43).

Four case series of esophageal GIST have reported on its features and survival (Table 2) (31,32,44,45). Lott et al. compared the prognosis of 55 cases of esophageal GIST with that of 683 cases of gastric GIST. Esophageal GISTs in these four case-series were generally classified as high-risk, and their prognosis in terms of OS was significantly worse than that of gastric GIST (HR 0.481, 95% CI 0.294–0.785, *P* = 0.003). There is still little evidence from clinical trials to support use of neoadjuvant imatinib for GIST (46). Downsizing GIST by preoperative administration of imatinib may contribute to better postoperative quality of life by preventing wide resection and preserving function. However, we need to be aware of the risk of rupture or bleeding as a result of tumor necrosis and cystic change (46). Kang et al. suggested that neoadjuvant imatinib can be used in patients with high mitotic rates and/or larger tumors to obtain negative microscopic (R0) resection and to reduce the risk of intraoperative complications, including tumor rupture (31). Nakano et al. (44) and Feng et al. (45) summarised the clinical outcomes of esophageal GIST reported in the literature. Nakano et al. summarised 153 patients, 139 (88%) of whom underwent surgery (44). Unlike esophageal carcinoma, esophageal GIST has tendency to recur at a constant rate after surgery. Of the 139 patients who underwent surgery, 23 (16.5%) developed recurrence, and metastatic disease was more common than local recurrence (18 vs. 5 patients). This indicates a need for a long-term follow-up in these patients. Feng et al. summarised 135 cases of esophageal GIST (45). The 5-year disease-free survival rate was 65.1% and tumor size was an independent prognostic factor. The most common site of distant metastasis was the liver, followed by the lung and thoracic cavity.

**Table 2:** Past reports on resectable gastrointestinal stromal tumors

**Authors**	**Patient inclusion period**	**N**	**Adjuvant therapy**	**Surgery**	**5-year DFS**	**5-year OS**
Kang et al. (31)	Oct 1996 to Dec 2015	27	0 (0%)	25 (93%)	–	–
Lott et al. (32)	2004 to 2012	55	6 (11%)	33 (60%)	65.3%	48.3%
Nakano et al. (44)	Jan 1983 to May 2011	153	–	139 (91%)	57.0%	88.7%
Feng et al. (45)	2000 to 2015	135	38 (28%)	125 (93%)	65.1%	–

Abbreviations: DFS, disease-free survival; OS, overall survival

There is little evidence of unresctable esophageal GIST. Global clinical guidelines for GISTs have been published from the European Society for Medical Oncology (47). However, there are no evidence or guidelines of esophageal GIST specifically. Under this circumstance, it is reasonable to follow the guideline in general. Imatinib therapy has been performed to unresectable or metastatic tumors (48). A randomised phase II trial of imatinib in patients with incurable GIST, B2222 trial, revealed that nearly 50% patients with advanced GIST treated with imatinib survived for more than 5 years (49). As described before, kinase genotype can predict the objective response and OS with patients treated with imatinib (50). KIT and PDGFRA wild-type GIST have no effective therapy options (51). Once the disease develops resistance to imatinib, second- and later-line options, including sunitinib and regorafenib, pimitespib, are applicable in the clinical practice. However, the response rate is low and clinical benefit is limited (52–54).

Although multidisciplinary treatment is desirable for esophageal GIST, the standard treatment is still a matter of debate. With more data from clinical trials involving large numbers of patients, we may be able to improve the prognosis of esophageal GIST in the future. At this point, when it comes to resectable esophageal GISTs, physicians need to consider whether enucleation is an option to achieve R0 resection to avoid deteriorated quality of life.

### Summary of esophageal GIST

Esophageal GIST is extremely rare and represents < 1% of all GIST cases. The key element for treatment is complete surgical resection and avoids intraoperative rupture. Enucleation or esophagectomy are the options for surgical resection and both strategies should be considered in each case due to the anatomical location of the tumor and the comorbidity of the patient. For unresectable esophageal GIST, molecular targeted drugs such as imatinib and sunitinib, regorafenib, pimitespib are widely used in the clinical practice based on the results of randomised controlled trials.

## Carcinosarcoma

### Epidemiology and diagnosis

Carcinosarcoma is a rare malignant tumor with carcinomatous and sarcomatous components (55). ESC is reported to account for 0.5–2.4% of all esophageal tumors (56) and occurs mainly in middle-aged and older men with a history of smoking, alcohol consumption and squamous cell carcinoma of the esophagus (55). In view of its low incidence, it has been difficult to establish a standard treatment for ESC. However, it is generally treated according to the protocols for EC (57). Three case series have reported on the treatment options used for ESC and their survival outcomes (Table 3) (58–60). According to these reports, esophagectomy has been the only potential curative therapy. Although there are some cases that have achieved a pathologically complete response (61,62), these cases are relatively rare. In ESC, the volume of the sarcomatous component is usually predominant over the squamous cell carcinoma component, indicating that ESC could be more resistant to chemotherapy or radiotherapy than squamous cell carcinoma EC (63).

**Table 3:** Previous case series on esophageal carcinosarcoma

**Authors**	**Patient inclusion period**	**N**	**Treatment**	**5-year survival rate**
Sano et al. (58)	–	20	OP (19/20) CT (1/20)	60%
Wang et al. (59)	Jan 2000 to Jan 2011	33	OP (23/33) OP + CT (4/33) OP + RT (3/33) RT + CT (1/33) TCM (2/33)	48%
Chen et al. (60)	Jan 2006 to Dec 2018	24	OP (12/24) OP + CT (3/24) OP + RT (1/24) OP + CT + RT (3/24) RT + CT (4/24) RT (1/24)	70.8%

Abbreviations: CT, chemotherapy; OP, operation; RT, radiotherapy; TCM, traditional Chinese medicine

### Treatments and future perspectives

The prognosis of ESC is controversial. Patients usually have symptoms of dysphagia at an early stage because ESC demonstrates a polypoid growth pattern and usually does not infiltrate deeply into the esophageal wall. Therefore, survival of patients with ESC is usually better than that of patients with SCC of the same size (64). However, Sano et al. (58) found that the 5-year survival rate was lower in patients with ESC than in those with ESCC when they compared only those with T1 disease (47.6% vs. 84.3%, *P* = 0.008). They attributed the poorer prognosis of ESC to the fact that the sarcomatous component metastasizes via the hematogenous route rather than the lymphatic route. Wang et al. (59) analyzed the outcomes in 33 patients with ESC treated at their institution and reported that their 5-year survival rate was better than that of patients with ESCC (48% vs. 34.2%). They also found that the 2-year progression-free survival rate was 50% and then reached a plateau. Furthermore, they performed a multivariate analysis and found that a higher preoperative neutrophil to lymphocyte ratio (NLR) was associated with significantly worse survival. The NLR could indicate tumor recurrence because it is a composite score that reflects host inflammatory activity, which promotes tumor progression. Therefore, they assumed that it may be acceptable to administer adjuvant treatment after curative resection in patients with a higher NLR. However, it must be noted that their study included a limited number of patients, and larger randomised trials are needed in the future. Chen et al. (60) investigated the characteristics of 24 patients with ESC from their institution and found a 5-year survival rate of 54.2%, which seems to be higher than that for patients with ESCC. Forty-two percent of their patients were diagnosed with stage T1 ESC and no lymph node metastasis. These patients had a 5-year survival rate of 90% which is extremely high. However, lymph node metastasis occurred in 57% of patients with stage T2–4 ESC, which suggests that multidisciplinary treatment may be necessary in these patients. In their study, patients who received chemoradiotherapy or radiotherapy alone had a 5-year survival rate of 60%, which indicates the possibility of a good pathological response. However, we still believe high-quality clinical trials are needed in the future to establish a high level of evidence regarding standard treatment for ESC. Although esophagectomy is the mainstream of treatment of ESC, we need to explore the risk factors of recurrence to identify the best candidates and the best timing to perform multidisciplinary treatment to achieve better prognosis.

The origin of ESC is not fully understood, although there is some evidence indicating that epithelial-mesenchymal transition could be responsible (63). One study found that most cases of ESC have a transitional zone between the carcinomatous and sarcomatous components and that both elements share the same genetic alterations (65). Moreover, in some cases, the sarcomatous elements reportedly express specific epithelial-mesenchymal transition markers (66,67).

Along with the need for prospective trials to develop a treatment strategy, tumor biology needs further study. With more specific data from a large number of patients, we could develop a better understanding of ESC.

### Summary of esophageal carcinosarcoma

ESC is generally treated according to the protocols for EC. Esophagectomy has been the only potential curative therapy because ESC is thought to be more resistant to chemotherapy or radiotherapy than squamous cell carcinoma EC. Either way, further study of tumor biology is expected to establish strong evidence and achieve better prognosis.

## Primary malignant melanoma of the esophagus

### Epidemiology and diagnosis

PMME is a rare and highly aggressive tumor with a high recurrence rate and poor prognosis. PMME accounts for 0.1–0.2% of all malignant esophageal tumors (68–70). The median age at diagnosis is 60.4 years, which is younger than that for esophageal squamous cell carcinoma (71). The incidence of PMME is significantly higher in men than in women, with a male-to-female ratio of approximately 2:1 (72,73). PMME originates in the lower to middle esophagus in approximately 90% of cases (71). It has been presumed that melanoblasts migrate from the neural crest to various sites, such as the epidermis, oral cavity and uvea, where the primary malignant melanoma arises (68,69,74,75). The esophagus normally does not contain melanoblasts, so abnormal migration of esophageal melanocytes may explain the occurrence of PMME (59,75).

Approximately 25–30% of PMMEs are accompanied by ‘melanocytosis,’ which is characterised by the presence of an increased number of pigment-laden melanocytes in the basal layer of the esophageal squamous epithelium and an increased quantity of melanin in the esophageal mucosa (69). The clinical manifestations are dysphagia, non-specific post-sternal pain, acid reflux and other gastrointestinal symptoms, which are similar to those of esophageal squamous cell carcinoma (71,72).

PMME is often detected or suspected based on endoscopic findings of a tumor that is elevated, well-circumscribed and pigmented. The tumor appears partially covered by normal mucosa and is rarely accompanied by ulceration. While a black tone is a known characteristic of PMME, it is important to conduct a careful evaluation for an accurate diagnosis. Approximately 10–25% of PMMEs show varying colors, including purple, brown and white, depending on the quantity of melanin. There is no melanin pigmentation in cases of ‘amelanotic melanoma.’ There are additional findings that support a definite diagnosis of PMME, including a sub-tumor referred to as a ‘satellite’ (which is believed to be an intramural metastasis), melanotic macules known as ‘melanocytosis’ and melanoma in situ (69).

Biopsies are carried out in approximately 70% of patients (69). Although there is a danger of triggering a hematogenous metastasis, there is no definitive evidence of a correlation between undergoing a biopsy and dissemination of cancer cells throughout the body. Consequently, it remains unclear whether there is a significant relationship between undergoing a biopsy and the likelihood of metastasis (76).

Despite any effort at biopsy, the accuracy of these diagnoses is not perfect, with misdiagnosis as poorly differentiated carcinoma in 20–50% of patients because of the absence of melanin granules (69,75,76).

Differential diagnosis of PMME and metastatic melanoma is challenging. PMME can be defined by identification of melanocytes at the epithelial-stromal junction and the absence of any other primary sites (75,77).

The criteria for diagnosis consist of two elements: (1) a distinctive histological pattern of melanoma, which contains melanin granules within the tumor cells, and (2) development in a region where there is junctional activity in the squamous epithelium. The term ‘junctional activity’ is defined as the presence of melanocyte clusters with different degrees of atypia at the mucosal-submucosal junction adjacent to the tumor mass (75,76).

### Molecular findings

NRAS and BRAF mutations are frequently observed in cutaneous melanoma (70,75,78). However, PMME sometimes has genetic alterations that are different from those of cutaneous melanoma. Somatic mutations in BRAF have been found in 60–70% of cutaneous melanomas (79) but in only a few cases of PMME (77). In contrast, the prevalence of NRAS and KIT mutations is higher in PMME than in cutaneous melanoma (70,78,79).

### Treatment and future perspectives

Given that there is no specific staging system for PMME, the eighth edition of the AJCC/UICC staging system for cancers of the esophagus and esophagogastric junction has been widely used (68,80).

Surgery has traditionally been the only option for prolonging survival in patients with PMME, and total or sub-total esophagectomy offer more favorable survival results (81). Since the efficacy of both chemotherapy and radiotherapy have been shown to be limited (72,77,81), it has been reported that approximately 60–80% of patients with PMME, for whom no other curative therapy is available and whose cancer has spread through the lymphatic vascular system, undergo surgery for locally resectable tumors (69,76,82).

There are several options for non-surgical treatment or adjuvant therapy with surgery for patients with PMME, including chemotherapy, radiotherapy and immunotherapy. However, none of these treatment options have shown significant efficacy when used as standalone treatment for this type of tumor (69,83).

Immune checkpoint inhibitors achieved significantly longer recurrence-free survival than placebo when used as adjuvant therapy for stage IIIB–D or stage IV melanoma in the CheckMate-238 trial (84,85). In that trial, the 4-year recurrence-free survival rate was 51.7% (95% CI 46.8–56.3) in the nivolumab group and 41.2% (95% CI 36.4–45.9) in the ipilimumab group (HR 0.71, 95% CI 0.60–0.86, *P* = 0.0003) (85).

The CheckMate-915 trial, which compared a combination of nivolumab plus ipilimumab with nivolumab alone as adjuvant therapy for stage IIIB-D or stage IV melanoma, failed to show any advantage of the combination therapy (86). In that trial, the 24-month recurrence-free survival rate was 64.6% in the combination therapy group and 63.2% in the nivolumab alone group (HR 0.92, 97.295% CI 0.77–1.09, p = 0.269) (86).

Both studies included a small number of patients with mucosal melanoma (84–86). However, the majority of cases involved patients with cutaneous melanoma. Although Nivolumab has demonstrated lower effectiveness in mucosal melanomas compared to cutaneous melanomas, certain studies have presented promising outcomes (72,81,87–89). However, the standard treatment and efficacy of ICI in PMME remain unclear due to its rarity. There is a requirement for randomised controlled trials involving larger cohorts of PMME patients (81).

The postoperative 5-year survival rate in patients with PMME has ranged from 4% to 37% (70,73,74). The increasing number of cases that are detected early has contributed to the improved prognosis (64).

In metastatic PMME, combined therapy of nivolumab plus ipilimumab tends to be selected as first-line therapy based on the CheckMate-067 trial (90). A pooled analysis of data for 35 patients with mucosal melanoma treated by nivolumab plus ipilimumab in the CheckMate-067 and CheckMate-069 trials indicated that the combination treatment was superior to nivolumab alone in terms of clinical efficacy, although the statistical significance of this finding was not reported (91). Two retrospective studies with larger sample sizes compared the efficacy of nivolumab plus ipilimumab with that of programmed cell death-1 inhibitor monotherapy (92). Both studies found that these treatments were similarly effective and concluded that addition of ipilimumab did not confer additional benefit over programmed cell death-1 inhibitor monotherapy in mucosal melanoma.

NRAS and KIT mutations are more frequently observed in PMME than in cutaneous melanoma. Clinical trials are currently in progress to identify the optimal treatment for melanoma with NRAS and KIT mutations. Considering that treatment strategies beyond second-line therapy are still controversial, these clinical trials may influence future treatment depending on the results.

### Summary of primary malignant melanoma of the esophagus

Primary malignant melanoma of the esophagus is a rare form of mucosal melanoma with a poor prognosis, which accounts for 0.1–0.2% of all malignant esophageal tumors. Surgery has stood as the sole recourse for extending the survival of PMME patients (Table 4) (72,77,81,93,94). In some case reports, the emergence of immunotherapy, particularly the integration of nivolumab, in conjunction with ipilimumab, has notably elongated the OS period for those with metastatic PPME.

**Table 4:** Past reports on primary malignant melanoma of the esophagus

**Authors**	**Patient inclusion period**	**N**	**Treatment**	**OS**	**RFS**
Wang et al. (72)	Jan 2008 to Sep 2017	76	Surgery 59/76 Adjuvant therapy 37/59	22.3 months	4.5 months
Hashimoto et al. (93)	Jan 1995 to Dec 2016	6	Surgery 4/6 ICI 2/6	19.6 months	19.3 months
Tae-Se Kim et al. (94)	2000 to 2020	17	Surgery 10/17 Chemmotherapy 5/17	10 months	4 months
Dai et al. (81)	Jan 1998 to Jan 2018	70	Surgery 70/70	13.5 month	5.9 month (DFS)
Lasota et al. (77)	/	16	Surgery 16/16	4–22 months	/

Abbreviations: RFS, recurrent-free survival; DFS, disease-free survival; OS, overall survival

## Conclusion

Rare ECs such as NEC, GIST, ESC and PMME have biological characteristics that differ from those of squamous cell carcinoma and adenocarcinoma. Therefore, different treatments are required. Immune checkpoint inhibitors and molecular targeted therapies have the potential to be effective in some of these rare ECs, but further investigations are needed to improve clinical outcomes.

## Data Availability

The corresponding author (Shun Yamamoto) should be contacted if someone requests the data of this review.
